# Molecular detection of *Anaplasma platys* infection in free-roaming dogs and ticks from Kenya and Ivory Coast

**DOI:** 10.1186/s13071-016-1443-3

**Published:** 2016-03-16

**Authors:** Ioana Adriana Matei, Gianluca D’Amico, Patrick K. Yao, Angela Monica Ionică, Paul W. N. Kanyari, Aikaterini Alexandra Daskalaki, Mirabela Oana Dumitrache, Attila D. Sándor, Călin Mircea Gherman, Moneeb Qablan, David Modrý, Andrei Daniel Mihalca

**Affiliations:** Department of Parasitology and Parasitic Diseases, Faculty of Veterinary Medicine, University of Agricultural Sciences and Veterinary Medicine Cluj-Napoca, Cluj-Napoca, Romania; Unité de Formation et de Recherche Biosciences, Université de Cocody, Abidjan, Côte d’Ivoire; Department of Veterinary Pathology, Microbiology and Parasitology, Faculty of Veterinary Medicine, University of Nairobi, Nairobi, Kenya; Department of Pathology and Parasitology, Faculty of Veterinary Medicine, University of Veterinary and Pharmaceutical Sciences, Brno, Czech Republic; CEITEC VFU, University of Veterinary and Pharmaceutical Sciences, Brno, Czech Republic; Institute of Parasitology, Biology Centre of Academy of Sciences of the Czech Republic, České Budějovice, Czech Republic

**Keywords:** Africa, *Anaplasma platys*, Carnivores, Kenya, Ivory Coast

## Abstract

**Background:**

*Anaplasma platys* is a bacterium parasitic in the canine platelets, representing the causative agent of canine cyclic thrombocytopenia, with a worldwide distribution, mainly in tropical countries. The agent has zoonotic potential, being reported in several human clinical cases. The suspected vector is the tick *Rhipicephalus sanguineus* (*sensu lato*), widely distributed in animals and humans in the tropical regions of South America, Africa, Asia and Australia, but also in southern Europe. Only few scattered data concerning the epidemiology of *A. platys* in sub-Saharan Africa are available. The aim of our study was to evaluate the epidemiological status of *A. platys* in dogs and cats from rural communities in eastern and western Africa, where dogs and their ticks live in close contact with humans.

**Methods:**

A total of 216 canine and 22 feline blood samples and ticks feeding on them were collected in 2013 and 2014 from eight localities in Ivory Coast and four localities in Kenya. PCR was performed using specific primers targeting a portion of the 16S rRNA gene, followed by sequencing.

**Results:**

The main results highlight the high prevalence of *A. platys* infection in dogs in both Eastern and Western Africa and report it for the first time in Eastern Africa and in *Rhipicephalus camicasi*.

**Conclusions:**

The presence of *A. platys* DNA in *R. camicasi* feeding on negative hosts together with the relatively high prevalence of *A. platys*, correlated with the absence of the probable vector *Rhipicephalus sanguineus* (*s.l*.) in Kenyan Island suggest the involvement of other tick species in the transmission of *A. platys*.

**Electronic supplementary material:**

The online version of this article (doi:10.1186/s13071-016-1443-3) contains supplementary material, which is available to authorized users.

## Background

*Anaplasma platys,* the agent of infectious canine cyclic thrombocytopenia (ICCT), is an obligate intracellular rickettsial organism of the family Anaplasmataceae [1, 2]. It is commonly infecting platelets where it forms basophilic morulae [1, 3]. *Anaplasma platys* infection was initially considered a subclinical condition with few or no clinical signs [1, 4, 5]. However, in studies performed in experimentally and naturally infected dogs in Greece [6], France [7] and Israel [8], clinical signs have been described, suggesting the involvement of more virulent *A. platys* strains [9]. Moreover, the zoonotic potential was suggested in the last few years in Venezuela, USA and Grenada or South Africa [10–12].

The first *A. platys* infection was described by Harvey et al. [1] in dogs from Florida, USA. Since then, the pathogen was recorded in several countries from the New World: Venezuela [13], Brazil [14], Chile [15], Argentina [16], Panama [17] and French Guiana [18]. In Europe it was reported in southern and western countries and in several countries from eastern and central Europe [19–25]. *Anaplasma platys* infection was reported in the majority of the countries from Asia and also in Australia [26–37].

In sub-Saharan Africa, *A. platys* was identified in *Rhipicephalus sanguineussensu lato* (*s.l*.) ticks collected from dogs in the Democratic Republic of Congo [38], in engorged ticks collected from domestic and wild ruminants in South Africa [39] and in dogs in Ivory Coast, Gabon and Nigeria [40, 41]. In North Africa, *A. platys* infection was also reported in ticks and dogs in Tunisia [42], Morocco [43] and Algeria [44].

Considering the scarce data regarding the epidemiology of *A. platys* in dogs in the sub-Saharan Africa, the almost ubiquitous presence of *R. sanguineus* (*s.l*.) on dogs and the zoonotic potential, the aim of this study is to evaluate the prevalence of *A. platys* and to extend the knowledge on its distribution in dogs from East and West Africa (Kenya and Ivory Coast), and to correlate its occurrence with ecological factors.

## Methods

### Study sites, blood sampling, tick collection and identification

Blood samples and ticks from dogs and cats were collected in 12 localities in Ivory Coast (April 2013) and Kenya (January 2014). A total of 216 free-roaming dogs and 22 cats were sampled. Demographic data, identification of animals (name, owner, age and sex), clinical signs and the presence of ectoparasites were recorded for each animal (Table 1). Blood was collected from the cephalic vein and then transferred into ethanol filled tubes for storage. Ticks were preserved in absolute ethanol. Morphological identification was done individually for each tick to the species level by using morphological keys and descriptions [45, 46].

**Table 1:** Prevalence of *Anaplasma platys* and ticks in dogs and cats

Collection sites	Mean age (month)	T	F	M	Prevalence (%)									
					*A. platys* (95 % CI)	Ticks	*Rsa*	*Rc*	*Rp*	*Rh*	*Rsi*	*Hl*	*Hy*	Am
Dogs														
Paté	13	10	5	5	10 (0.25–44.5)	80	0	60	60	20	0	10	0	0
Mtanga Wanda	21	9	7	2	22.2 (2.81–60.01)	100	0	66.7	55.6	11.1	0	0	0	11.1
Kizingitini	27	22	8	14	22.7 (7.82–45.37)	95.5	0	95.5	27.3	0	0	0	0	0
Matondoni	23	45	27	18	17.8 (8–32.05)	71.1	2.2	67.7	22.2	0	2.2	0	0	4.5
**Kenya**	**26**	**86**	**47**	**39**	**18.6 (11.02–28.45)**	**81.4**	**1.2**	**73.3**	**31.4**	**3.5**	**1.2**	**1.2**	**0**	**3.5**
Abidjan	34	38	18	20	0	14.3	0	0	0	0	0	14.3	0	0
Bieby	17	15	4	11	0	86.7	26.7	0	0	0	0	60	0	0
Taabo	31	21	7	14	0	66.7	33.3	0	0	0	0	23.8	4.8	4.8
Mgbankagouti	20	4	3	1	0	100	50	0	0	0	0	50	0	0
Zoukoussi	15	18	9	9	22.2 (6.41–47.64)	88.9	77.8	0	0	0	0	11.1	0	0
Irobo	12	23	12	11	30.4 (13.21–52.92)	95.7	78.3	0	0	0	0	17.4	0	0
Gboyo	43	21	11	10	0	0	0	0	0	0	0	0	0	0
** Ivory Coast**	**26**	**140**	**64**	**76**	**8.5 (4.3–14.64)**	**56.2**	**37.7**	**0**	**0**	**0**	**0**	**16.9**	**0.8**	**0.8**
Cats														
Matondoni	44	15	6	9	0	0	0	0	0	0	0	0	0	0
Taabo	13	7	4	3	0	14.3	14.3	0	0	0	0	0	0	0

Legend: *Rsa*: *R. sanguineus* (*s.l*.); *Rc*: *R. camicasi*; *Rp*: *R. pulchellus*; *Rh*: *R. humeralis*; *Rsi*: *R. simus*; *Hl Haemaphysalis leachi*; *Hy*: *Hyalomma* sp.; *Am*: *Amblyomma *sp; In Bold: overall prevalence by Countries

**Fig. 1:**
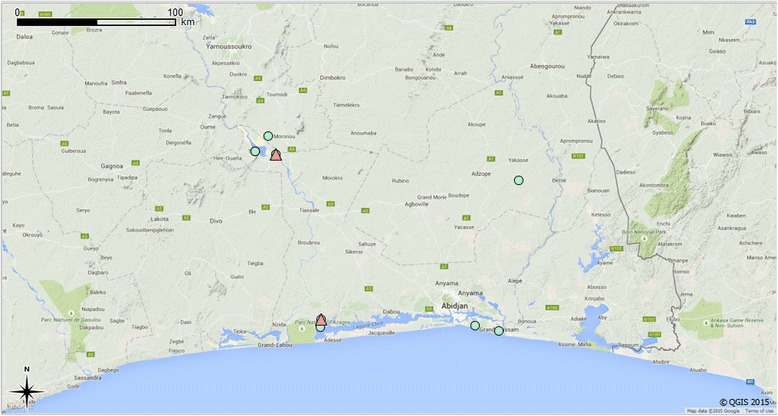
Collection sites and *A. platys-*positive sites from Ivory Coast. ● collection sites; ▲ *A. platys-* positive sites

**Fig. 2:**
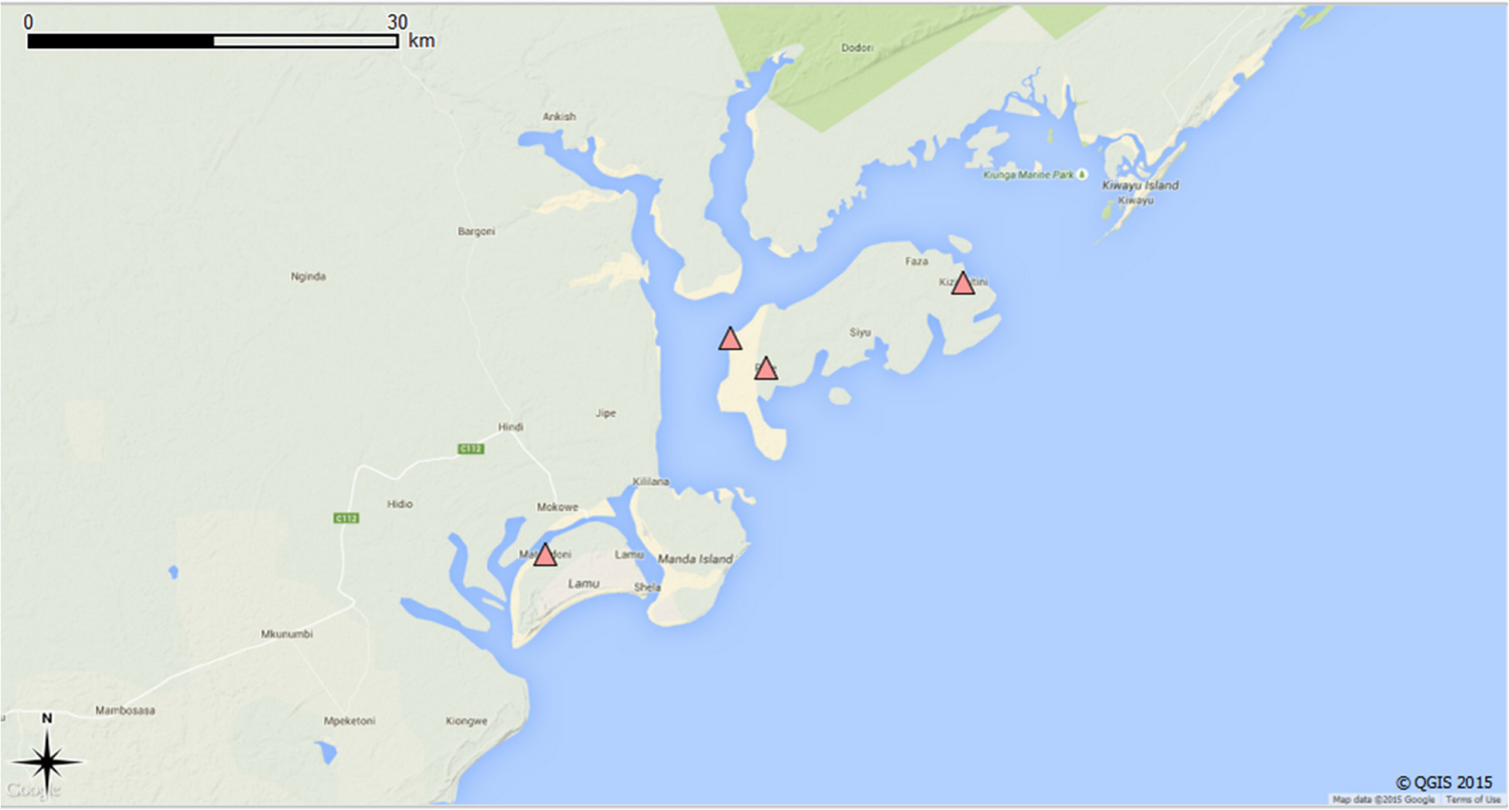
Collection sites and *A. platys-*positive sites from Kenya. ● collection sites;▲ *A. platys-*positive sites

In Ivory Coast, the sampling sites (*n =* 8) were located in the southern part of the country (5.0–5.5 N/3.5–5.5 W°, Fig. 1, Additional file 1: Figure S1), while in Kenya the samples originated from four locations situated on Paté and Lamu Islands in Lamu Archipelago (2.0–3.35 S/40.8–41.15 E°, Fig. 2, Additional file 2: Figure S2). The collection sites from Ivory Coast belong to Eastern Guinean Forest ecoregion (Tropical and Subtropical Moist Broadleaf Forest), the dominant biome being semi-deciduous and moist evergreen forests [47]. The temperature ranges between 22 and 34 °C. The rainfall pattern can be divided into two wet and dry seasons, with a rainfall average between 1,400 and 2,500 mm per year [47].

Lamu and Paté Islands (Kenya) belong to the Eastern African Mangroves ecoregion. Annual rainfall averages range between 750 and 1,500 mm with two distinct rainy seasons, the temperatures are high, ranging between 23 and 33 °C [48].

### DNA extraction

Genomic DNA was extracted from ethanol preserved blood clots and ticks using a modified phenol-chloroform method [49, 50]. Crushed ticks and 200 μl of clotted blood were dried at 56 °C for 30 min and then suspended in 1.5 ml lysis buffer (0.1 M NaCl, 0.05 M EDTA, 0.01 M Tris, 4.8 % SDS; pH 8). The digestion was performed in 20 μl proteinase K (Bioline, UK) at 56 °C for 90 min for blood and respectively overnight for ticks. After protein lysis, the mixture was extracted with an equal volume blend of phenol and chloroform, followed by one extraction with chloroform alone. Each extraction step included 1 min of shaking and 10 min centrifugation (13,000 × g). DNA was precipitated with 96 % ethanol for 15 min and then dried at room temperature in sterile medium for 12 h. The dried DNA pellet was re-suspended by adding 100 μl of PCR water. For each extraction procedure, negative controls consisting in reaction mixes without DNA (PCR water instead of blood) were used, in order to check for possible cross-contamination. Isolated genomic DNA from a representative number of samples was assessed quantitatively by Nanodrop ND-1000 spectrophotometer analyzer (NanoDrop Technologies, Inc., Wilmington, DE, USA).

### Polymerase chain reaction (PCR) and agarose gel electrophoresis

PCR was initially performed using a group-specific set of primers that amplify a 345 bp fragment of the 16S rRNA gene. The primer sets EHR16SD (5′-GGTACCYACAGAAGAAGTCC-3′) and EHR16SR (5′-TAGCACTCATCGTTTACAGC-3′) amplify various species including *Ehrlichia canis, E. chaffeensis, E. muris, Cowdria ruminantium, Anaplasma phagocytophilum, A. platys, A. marginale, A. centrale, Wolbachia pipientis, Neorickettsia sennetsu, N. risticii,* and *N. helminthoeca* [51]. For all positive samples, a second PCR reaction was carried out using *A. platys* specific primers EPLAT5/EPLAT3 (forward primer: 5′-TTTGTCGTAGCTTGCTATGAT-3′ and a reverse primer: 5′-CTTCTGTGGGTACCGTC-3′), amplifying a 349-bp fragment of the 16S rRNA gene [52]. The amplification was performed as follows: 25 μl reaction mixture containing 4 μl aliquot of isolated DNA, 12.5 μl of 2× Green Master Mix (Rovalab GmBH) and 1 μl of each primer (0.01 mM). The PCR was carried out using the T100^TM^ Thermal Cycler (Bio-Rad). The amplification profile of the first PCR consisted of 5 min of initial denaturation at 95 °C, followed by 35 cycles of denaturation at 94 °C for 30 s, annealing at 55 °C for 30 s and extension at 72 °C for 90 s, and a final extension at 72 °C for 5 min. The protocol used for the second PCR was: 5 min of initial denaturation at 95 °C, followed by 40 cycles of denaturation at 94 °C for 30 s, annealing at 58 °C for 30 s and extension at 72 °C for 45 s and a final extension at 72 °C for 5 min. In each PCR reaction set (48 samples), a positive and a negative control were included in order to assess the specificity of the reaction and the possible presence of contaminants. Positive controls consisted of DNA isolated from the blood of a dog naturally infected with *A. platys* in Israel. The reaction mix without DNA was used as a negative control. Amplicons were visualised by electrophoresis in a 1.5 % agarose gel (1 × TAE, pH 8.0) stained with SYBR® Safe DNA gel stain (Invitrogen) and their molecular weight was assessed by comparison to a molecular marker (O’GeneRuler™ 100 bp DNA Ladder, Thermo Fisher Scientific Inc., USA). All *A. platys* positive PCR samples were sequenced.

### DNA sequencing

PCR products were purified from amplicons using the QIAquick PCR Purification Kit (QIAGEN). Sequencing analysis was performed (Macrogen Europe, Amsterdam) and the obtained sequences were compared with those available in GenBank^TM^ by Basic Local Alignments Tool (BLAST) analysis.

### Statistical analysis

Statistical analysis was performed using EpiInfo^TM^7 (CDC, USA) software. The total infection prevalence of *A. platys* (95 % CI), the infection prevalence based on age and sex of dogs, the prevalence of *A. platys* DNA presence in each ticks species, the infection prevalence in each location and the infection prevalence characterised by presence or absence of ticks on the animals and by ecoregions was assessed using the chi-square independence test. Maps showing the collection sites and the positive locations for *A. platys* were generated using QGIS 2.6 software.

### Ethics statement

This study was approved by the USAMV CN Bioethics committee with the registration number 23/21–09–2015, following the EU 2010/63 and National directives Ord. 28/31–08–2011 and National Law 206/2004. Our projects developed in these countries followed the respective national laws, they were performed with the owners informed consent and were supervised by local Veterinary Authorities.

## Results

A total of 238 blood samples from 216 dogs and 22 cats from Kenya and Ivory Coast were analysed for the presence of *A. platys* DNA using PCR. The overall prevalence in dogs was 12.5 % (27/216, 95 % CI: 8.4–17.66). All blood samples collected from cats were negative. In Kenya, the bacterium was identified in all four locations, while in Ivory Coast it was present in two out of eight locations. In the positive locations, the prevalence of infection varied between 10 and 30.4 %, with an average value of 21.26 % (27/127; 95 % CI: 14.5–29.4). The highest prevalence was recorded in Irobo village (Ivory Coast). When considering all collection sites, the differences in prevalence between localities were statistically significant (*χ*^2^ = 25.11, *df* = 11, *P  <* 0.01). However, no significant difference was observed when considering only the positive localities (*χ*^2^ = 2.28, *df* = 5, *P  >* 0.05). The prevalence of *A. platys* infection in each locality is shown in Table 1.

The overall prevalence by countries and respectively by ecoregions was significantly higher (*χ*^2^ = 4.86, *df* = 1, *P  <* 0.05) in Kenya compared to Ivory Coast (Table 1).

The prevalence of infection in young dogs (under 1 year) was 19.79 % (19/96, 95 % CI: 13.36–29.17) and the prevalence in adult dogs was 6.67 % (8/120, 95 % CI: 2.92–12.71); the young dogs were significantly more infected (*χ*^2^ = 8.4, *df* = 1, *P  <* 0.005). The prevalence of *A. platys* infection in females was 10.89 % (11/101, 95 % CI: 5.56–18.65) and in males 13.91 % (16/115, 95 % CI: 8.17–21.61), without significant differences between them.

Overall, 65.74 % (142/216, 95 % CI: 59–72.1) of the dogs were infested by ticks; of these, 16.9 % (24/142, 95 % CI: 11.14–24.10) were also positive for *A. platys*; only 4.05 % (3/74, 95 % CI: 0.84–11.39) of the dogs that were free of ticks were positive, the difference between these two categories being statistically significant (*χ*^2^ = 7.3, *df* = 1, *P  <* 0.05).

The prevalence of *A. platys* DNA presence in *R. sanguineus* (*s.l*.) was 4.38 % (16/365, 95 % CI: 2.61–7.17) in respectively 1.62 % (6/371, 95 % CI: 0.66–3.66) in *R. camicasi*. The other identified tick species shown in Table 1 were negative for *A. platys* DNA. Out of 16 *A. platys-*positive ticks, ten *R. sanguineus* (*s.l*.) derived from *A. platys-*positive dogs (6.49 %, 10/154, 95 % CI: 3.16–11.62) while six derived from negative dogs (2.84 %, 6/211, 95 % CI: 1.05–6.09) (Table 2). The positive *R. camicasi* were also collected from both positive and negative dogs, with a prevalence of *A. platys* DNA presence of 6.15 % (4/65, 95 % CI: 1.7–15.01) in ticks derived from positive dogs and 0.65 % (2/306, 95 % CI: 0.11–2.6) in ticks collected from negative dogs (Table 2).

**Table 2:** Prevalence (in %) of *Anaplasma platys* in ticks related to the host infection status

*A. platys*	Negative ticks		Positive ticks	
	*R. camicasi*	*R. sanguineus*	*R. camicasi*	*R. sanguineus*
Negative dogs (Kenya)	99.35	0	0.65	-
Positive dogs (Kenya)	93.85	-	6.15	-
Negative dogs (Ivory Coast)	-	97.16	-	2.84
Positive dogs (Ivory Coast)	-	93.51	-	6.49

The data regarding the structure of tick populations and the prevalence of each tick species are presented in Table 1. In Ivory Coast the presence of *A. platys* in dogs from one area can be correlated with the high prevalence (> 50 %) of *R. sanguineus* (*s.l*.) (Tabel 1, *k* = 1) and in Kenya, with the high prevalence (> 50 %) of *R. camicasi* (Table 1, *k* = 1).

From 49 *A. platys-*positive samples were obtained 37 sequences with a length varying between 320 and 340 bp. The BLAST analysis of all sequences showed 99 to 100 % similarity with various *A. platys* strains isolated from dogs originating from South America, Asia and Africa (accession numbers: KC989957, KF360842, JX112780, GQ395384,KC109446, KF576217, JX112781, AF478131 and KT357373). All sequences obtained from dogs and ticks were highly similar (99.7 %) amongst each other.

## Discussion

This study reports the presence of *A. platys* in dogs from Ivory Coast and Kenya. This is the first report of this pathogen in dogs from East Africa and the first evidence of *A. platys* in *R. camicasi* ticks. Few reports are available regarding the presence of *A. platys* in Africa.

The overall prevalence of *A. platys* infection obtained in our study is higher than in other previous studies from Africa, but falls within the global average. The prevalence obtained in Kenya is similar to the highest prevalence rates, which were recorded in Brazil [53], Venezuela [54] and Japan [55]. The prevalence based on age groups (juveniles and adults) obtained in our study was higher in juvenile dogs (under 1 year-old). Similar results were obtained in the study of Brown et al. [56]. However, other studies showed no significant differences [41, 53]. Our results could be influenced by the tick burden, which is higher in young dogs [57, 58], including the ones sampled in this study (data not shown).

The different results regarding the prevalence in Ivory Coast and Kenya could be explained by the unequal sampling effort (140 *versus* 86), by the different collection time and by the presence and abundance of the tick vector. Besides molecular detection of *A. platys* in this tick species, the geographical distribution of *A. platys* infection seems to overlap with the distribution of the brown dog-tick *R. sanguineus* (*s.l*.), as described by Walker et al. [45]. Despite these facts, an experimental study failed to demonstrate the vectorial capacity of this tick species [59]. The employed microscopy method used for diagnosis showed a low sensitivity in experimental models [56]. However, *R. sanguineus* (*s.l*.) is still considered the most probable vector [38, 41].

The presence of *R. sanguineus* (*s.l*.) in Africa was described by Cumming [60] and Walker et al. [42]. In tropical and subtropical regions *R. sanguineus* s.l. is active throughout the year [61, 62] and apparently has no seasonality [62, 63]. However, a warmer temperature may contribute to increased tick abundance by a more rapid development resulting in lower mortality rates [64]. The abundance of the vector could influence the presence of *A. platys* because of the short bacteremia. In experimental models the average PCR positivity was 109 days after infection [65].

The prevalence of *A. platys* infection could be influenced also by the structure of the tick community, which was different in the collection sites, suggesting an influence of the environmental factors. The low prevalence or even absence of *R. sanguineus* (*s.l*.) in the sampled localities from the Kenyan islands and the presence of *A. platys* DNA in *R. camicasi* collected from negative hosts, suggest the possibility of this tick species acting as vector in this area. This hypothesis may be sustained by other studies that reported the presence of *A. platys* in other species of *Rhipicephalus*, such as unengorged *R. turanicus* in Israel and *R. evertsi* in South Africa [39, 66]. Moreover, *Anaplasma* strains closely related to *A. platys* were isolated from *R. bursa* in Turkey [67]. Further research on the topic is needed in order to confirm the vectorial role of other tick species.

The discrepancy between the high prevalence of *A. platys* infection in Kenyan dogs and the low prevalence in *R. camicasi* collected from negative hosts suggests the involvement of others factors such as the unequal number of the tested dogs and tick populations and the unknown previous hosts of *R. camicasi* during the immature stages [45].

Although in our study we did not find *A. platys* infections in the feline samples, there are two reports of infection in cats [3, 68] from Brazil and Thailand.

The zoonotic potential of *A. platys* is known, but its public health impact is still under debate. In the last few years, three human cases of multiple infections including *A. platys*, with clinical signs were reported [10, 11]. Also two clinical cases with single *A. platys* infection were recently confirmed in two women from Venezuela [12].

## Conclusions

The results of this study highlight for the first time the presence of *A. platys* infection in dogs originating from East Africa. Moreover the detection of *A. platys* DNA in *R. sanguineus* (*s.l*.) ticks from Ivory Coast sustains its probable vectorial role. However presence of *A. platys* DNA in *R. camicasi* correlated with absence of the probable vector *R. sanguineus* (*s.l*.) in Kenyan Island suggest the involvement of other tick species in the transmission of *A. platys*.

## Additional files

Additional file 1: Figure S1. klm. Geographic coordinates of collection sites from Ivory Coast. (KML 4 kb) Additional file 2: Figure S2. klm.Geographic coordinates of collection sites from Kenya. (KML 1 kb)
